# *In silico* gene knockout prediction using a hybrid of Bat algorithm and minimization of metabolic adjustment

**DOI:** 10.1515/jib-2020-0037

**Published:** 2021-08-04

**Authors:** Mei Yen Man, Mohd Saberi Mohamad, Yee Wen Choon, Mohd Arfian Ismail

**Affiliations:** School of Computing, Faculty of Engineering, Universiti Teknologi Malaysia, Skudai, Johor, Malaysia; Department of Genetics and Genomics, College of Medical and Health Sciences, United Arab Emirates University, Al Ain 17666, Abu Dhabi, United Arab Emirates; Institute for Artificial Intelligence and Big Data, Universiti Malaysia Kelantan, Kota Bharu 16100, Kelantan, Malaysia; and Department of Data Science, Universiti Malaysia Kelantan, Kota Bharu 16100, Kelantan, Malaysia; Faculty of Computing (FKOM), College of Computing and Applied Sciences, Universiti Malaysia Pahang, Lebuhraya Tun Razak, 26300 Gambang, Kuantan, Pahang, Malaysia; Department of Data Science, Universiti Malaysia Kelantan, Kota Bharu 16100, Kelantan, Malaysia; Faculty of Computing (FKOM), College of Computing and Applied Sciences, Universiti Malaysia Pahang, Lebuhraya Tun Razak, 26300 Gambang, Kuantan, Pahang, Malaysia

**Keywords:** Bat algorithm, bioinformatics, *Escherichia coli*, gene knockout, lactate, minimization of metabolic adjustment, succinate

## Abstract

Microorganisms commonly produce many high-demand industrial products like fuels, food, vitamins, and other chemicals. Microbial strains are the strains of microorganisms, which can be optimized to improve their technological properties through metabolic engineering. Metabolic engineering is the process of overcoming cellular regulation in order to achieve a desired product or to generate a new product that the host cells do not usually need to produce. The prediction of genetic manipulations such as gene knockout is part of metabolic engineering. Gene knockout can be used to optimize the microbial strains, such as to maximize the production rate of chemicals of interest. Metabolic and genetic engineering is important in producing the chemicals of interest as, without them, the product yields of many microorganisms are normally low. As a result, the aim of this paper is to propose a combination of the Bat algorithm and the minimization of metabolic adjustment (BATMOMA) to predict which genes to knock out in order to increase the succinate and lactate production rates in *Escherichia coli* (*E. coli*).

## 1 Introduction

The concept of using cell factories in the manufacture of conventional products such as alcohol, wine, pizza, and yoghurt, as well as a variety of pharmaceuticals such as antibiotics, hormones, and anti-cancer medications, is well-known [1]. Nevertheless, more recently, there has also been much focus on using cell factories to produce fuels and biochemicals such as bioethanol as a classic example, citric acid going into soft drinks, and more recently, 1,3 propanediol that is used for polymer production [2]. In the biorefinery concept, biomass that is converting into sugars can come from different sources. The sugars are then fermented by microorganisms to produce fuels and chemicals. The cell factory is used for this bioconversion process. Metabolic engineering is used to this cell factory in order for it to change its metabolism so it can produce different fuels and chemicals. The development of the cell factory used in the biotransformation is a research-intensive part, and the total cost for developing a cell factory is typically in the range of $50 million US dollars. By using genetic engineering, new pathways can be inserted into the strain of microorganism. However, the development of the proof of principle strain to the final strain that meets specific yield, titer, or productivity takes 3–6 years to complete. Novel technologies are needed to reduce the development time so the new products and new technologies can be brought onto the market faster [3].

Computational models are set up with various mathematical representations of metabolism, metabolic pathways, metabolic elements, enzymes, and metabolites in order to provide a simplistic view of reality. In the development and evaluation of synthetic cell biology in novel cell factories, these *in silico* quantitative measurements of variables are useful [4]. Computational approaches are introduced to reduce the time and cost of the development of the cell factory. OptKnock is one of the tools that was first developed to try to implement an optimal knockout framework where the idea is how the fluxes are optimally distributed when there is a gene knockout [5]. However, Optknock has the disadvantage of producing impractical knockout strategies due to insufficient restrictions on the resulting flux distributions. The minimization of metabolic adjustment (MOMA) assumption, on the other hand, provides more stringent phenotypic constraints to steady-state fluxes for engineered knockout strains with validated congruency with experimental findings, and it may provide stronger constraints to knockout steady-state flux distributions in order to systematically look for more practical knockout strategies in a metabolic network. Brochado and colleagues [6] developed the minimization of metabolites balance (MiMBl) as an alternative to MOMA, with the goal of overcoming some of its drawbacks. Instead of addressing the problem by identifying linear combinations of fluxes, MiMBl focuses on metabolite turnovers, which eliminates issues related to the sensitivity of the solutions to stoichiometric representations, which can have a significant impact on phenotypic predictions. MOMAKnock is introduced to improve the performance of OptKnock [7]. MOMAKnock introduced a bi-level programming problem to determine optimal metabolic genes or reactions to knockout for maximizing the target chemicals. Besides, several optimization algorithms such as Harmony Search, Artificial Bee Colony, Particle Swarm Optimization, and Cuckoo Search were introduced to hybridize with MOMA [8–11]. In this paper, the applicability of MOMAKnock is extended by formulating the *in silico* design problem by using Bat algorithm, hereafter referred to as BATMOMA. Bat algorithm is based on the echolocation behavior of microbats [12]. Microbats are one of the types of bat that are small to medium-sized (weighing from 3 to 150 g with wingspans around 25 cm) and eat insects. Microbats use a sonar that is called echolocation to detect prey. Microbats will create a high pulse of highly pitched sound. The echo will bounce back to tell microbats about the distance, the size, and how fast the target is. Bat algorithm is proven to be efficient in solving multi-stage, multi-machine, multi-product, scheduling problems, and the NP-hard (non-deterministic polynomial-time hardness) problems.

In this paper, BATMOMA is proposed, and the results of two case studies where *Escherichia coli* (*E. coli*) as the target microorganism is presented. The performance of BATMOMA is evaluated and compared with previous works within experimental approaches. Future work is suggested in the last section of this paper.

## 2 Methodology

### 2.1 Minimization of metabolic adjustment (MOMA)

**Figure 1:**
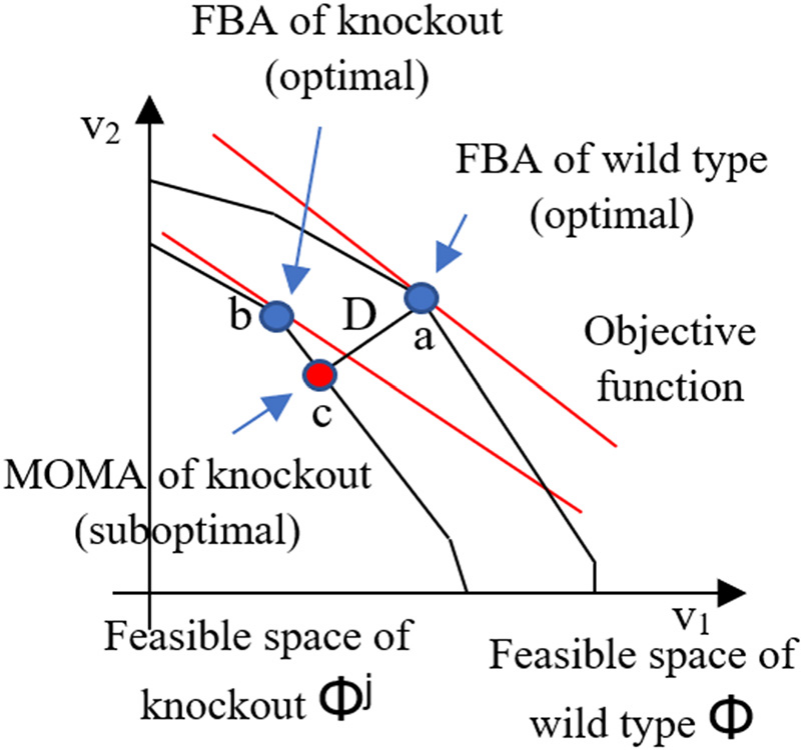
The optimization principles underlying FBA and MOMA [13].

To boost FBA flux calculation of knocked-out organism phenotypes, the minimization of metabolic adjustment (MOMA) method was developed. MOMA has similar stoichiometric restrictions to FBA, but for gene knockouts or deletions, MOMA modifies the optimal growth flux principle. At first, MOMA’s flux distribution between wild-type and mutant optimums is likely to be suboptimal. In addition, MOMA investigates this issue by bringing to the test the hypothesis that a gene deletion results in minimal flux redistribution as compared to wild-type metabolism. MOMA is a more accurate tool for estimating gene knockout bacteria’s metabolic phenotype. Figure 1 illustrates optimization principles underlying FBA and MOMA. The schematic two dimensional of Figure 1 depicts the feasible space for the wild-type (Φ^wt^) represented by green polygon and feasible space of mutant for flux *j* (Φ^*j*^) represented by superimposed yellow polygon. The coordinates are both two arbitrary representative fluxes, which is a simpler representation of multidimensional flux space. The FBA optimal prediction is the wild-type (a) and knockout (b). Regardless, MOMA uses quadratic programming to measure the substitute MOMA solution (c), which can be thought of as an approximation of the FBA optimum into the mutant’s feasible space. As a result, there is a unique solution for mutant FBA and MOMA [13].

MOMA, in fact, is unable of predicting optimal growth or metabolic functions. To estimate the metabolic phenotype, it measures the distance minimization in flux space (as shown in Figure 1). MOMA uses quadratic programming to decide which point in the flux space is nearest to the wild type point, consistent with the gene deletion constraint, or matches the gene deletion constraint [13].

### 2.2 A hybrid of Bat algorithm and minimization of metabolic adjustment (BATMOMA)

**Figure 2:**
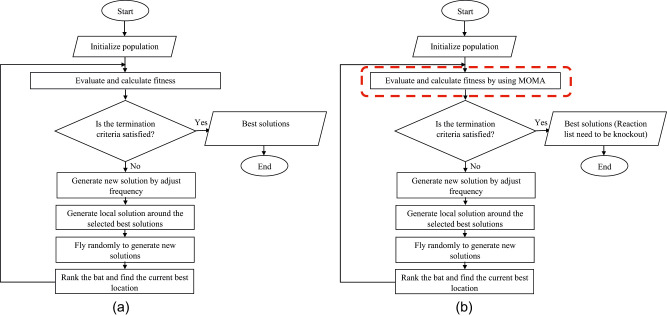
(a) Flow chart of Bat algorithm. (b) Flow chart of BATMOMA. The dotted box represents the proposed MOMA that is hybridized into Bat algorithm.

This paper introduces a new hybrid algorithm, BATMOMA, and this section will explain the details of BATMOMA. Figure 2(a) shows the flow chart of Bat algorithm, and 2(b) shows the flow chart of BATMOMA. The main difference between Bat algorithm and BATMOMA is the fitness evaluation where BATMOMA adapts MOMA into the fitness evaluation process. All bats have a frequency, position, velocity, loudness, and pulse rate. Sound waves used in this algorithm are created by microbats to detect their prey. All bats use echolocation to sense distance, and they also know the difference between prey and background barriers. Next, position means a place where something is located, the microbats’ position is denoted with *X*_*i*_, and velocity means the speed of something in a given direction. In this algorithm, the microbats have the velocity in the direction of their target. Then, loudness means the characteristic of the sound; how loud or soft the sound seems to its listeners, it is denoted with *A*_*i*_; pulse means a wave or vibration and is denoted with *r*_*i*_. Bats fly randomly with velocity *V*_*i*_ at position *X*_*i*_ with different frequency ranges *f*[min, max], varying wavelength and loudness *A*_*i*_ to search for prey. They can automatically adjust the wavelength of their emitted pulses and adjust the pulse emission rate depending on their target proximity. The loudness varies from a large *A*_*i*_ to a minimum constant value *A*_min_.

**Figure 3:**
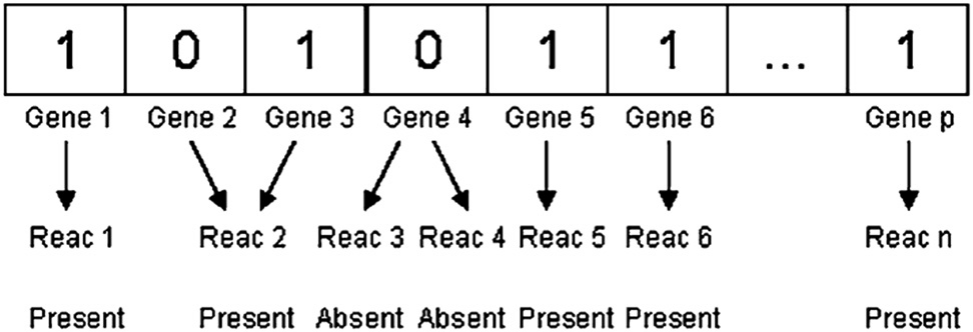
The representation of bat representation of metabolic genotype. Reac represents reaction.

In each reaction of the metabolic model, one or more genes are expressed by a binary variable. While a gene is absent, it is assigned a value of 0; when the gene is present, it is assigned a value of 1. A bat is produced at random from these variables, showing a rare mutation in which certain genes in the reaction are absent as compared to the wild type. Figure 3 depicts a metabolic genotype representation in the form of a bat.

The parameters are defined before the process started. *n* (population size) is set to 20, representing 20 bats. All bats have frequency, position, loudness, velocity, and pulse rate. Initially, random values for frequency, position, loudness, velocity, and pulse rate are assigned to each bat. Each bat is associated with a velocity and a position in the search space at each iteration, with respect to frequency.

Next, the population is evaluated using MOMA. The objective function is to maximize target chemical’s production rate in *E. coli* after gene knockout. The algorithm generates two values after the calculation, growth rate and production rate. The growth rate is used to decide if the cell survives after knocking out the genes. Hence, growth rate must be greater than 0.1 h^−1^, and production rate must be greater than 0.001 mmol gDW^−1^ h^−1^ to avoid a small value deemed to be significant improvement.

In Bat algorithm, a bat’s movement is controlled by two flight modes. The first mode (also known as the global search step) is the guided flight mode, where all bats are guided toward the bat with the best location (the solution with the best fitness value). The second flight mode is known as the local search step, where a new location for each bat is generated locally.

Before moving into global step, the echolocation parameters of all bats are initialized. The *Ngen* (number of generations) is set to 50. If the *Ngen* is 1, *Ngen* is less than the maximum number of iterations, the condition is true, then a new solution will be generated by adjusting the frequency and updating the velocity and position of the first bat. At each iteration, frequency, velocity, and position can be updated as per the following equation:

(1) $$f_{i}=f_{\min}+\left(f_{\max}-f_{\min}\right)\beta$$

(2) $$v_{i}^{t}=v_{i}^{t-1}+\left(x_{i}^{t}-x*\right)f_{i}$$

(3) $$x_{i}^{t}=x_{i}^{t-1}+v_{i}^{t}$$

First, current frequency would be calculated. Current frequency (*f*_*i*_) is equal to the minimum frequency (*f*_min_) plus maximum frequency (*f*_max_) minus minimum frequency into *β*. *β* is a random value that ranges between 0 and 1. *x*∗ is the current best position.

Next, the condition will be checked if the pulse rate with random value is greater than the pulse rate. The random value ranges between zero and one. If the condition is true, a solution among the best solution will be selected. The solution is selected according to the frequency as the waves’ frequency increases when the bat finds its prey. It means that a rich source of food is close to the selected bats in the surrounding neighborhood, and the other bat will fly to this region.

After the solution is selected, a local solution around the best solution is generated, and the position is updated using Eq. (4).

(4) $$x_{new}=x_{old}+\varepsilon A^{t}$$

The new position is calculated by adding the old position value to random number (*ε*) range −1 to 1 and the bat’s average loudness, *A* ^*t*^. Next, if loudness with random value is less than the loudness is checked. Loudness decreases as the bat move closer to the prey, and the pulse rate emission increases. If the condition is true, the solution is accepted, the pulse rate is increased, and the loudness is decreased. Equation (5) is used to increase the pulse rate.

(5) $$A_{i}^{t+1}=\;\propto A_{i}^{t},\;r_{i}^{t+1}=r_{i}^{0}\left[1-\exp\left(\gamma -t\right)\right]$$

The next step is to rank the bat. The loudness, pulse rate, and frequency are checked. According to that, the bat that has the highest frequency is the current best solution. It means that the bat will fly toward the leader to compete for food if the food source became scarce and does not exist around the randomly selected bat. These steps are repeated until a stopping criterion is met, which is either the maximum loop value is met or the fitness function has converged.

### 2.3 Experimental setup

MOMA is integrated into Bat algorithm in by using MATLAB. MATLAB is used to analyse data, generate models and applications, design algorithms, visualisation, and numerical computation. MATLAB was chosen because it is faster than many traditional programming languages, such as C++, and also it facilitates the execution of COBRA Toolbox. A workstation with at least a 2.1 GHz quad-cores processor, a dual-cores graphics processing unit, and 4GB RAM are required to carry out the simulation. The source code of BATMOMA can be downloaded through https://drive.google.com/file/d/1U0VP6E8932yNlK_gT4CGPNkDb1wgi1Oa/view?usp=sharing. The *E. coli* dataset is used in this paper for simulation and can be obtained through http://systemsbiology.ucsd.edu/Downloads/EcoliCore. This model is chosen because it is adaptable to any computing medium (such as MATLAB or Mathematica). The model is on a small scale, but it contains enough reactions and mechanisms to allow for interesting and informative simulations, while still being straightforward enough that the effects of such calculations can be readily understood. The simulations were performed for aerobic minimal media conditions where the glucose uptake rate was fixed to 10 mmol/gDW/h and the maintenance ATP constraint of 7.6 mmol ATP/gDW/h. Comparative results with the previous works are reported in this paper [5, 6, 9]. The unit of concentration is millimole (mmol), and the unit measurement in the experiments is millimoles per hour (mmol/h). The results presented are the best result after 50 individual runs.

## 3 Results and discussion

This section presents the experimental and comparative results into three sub-sections. The results are tabulated into six tables and illustrated into two figures. The experimental results are compared with the previous works, the effects of the knockout are validated with biological literatures. The initial growth rate for the model is 0.9219 h^−1^. The maximum theoretical rate for succinate of the wild-type *E.coli* model is 17.1429 mmol gDW^−1^ h^−1^ and 20 mmol gDW^−1^ h^−1^ for lactate.

### 3.1 Experimental and comparative results for succinate

The experimental result obtained from BATMOMA is displayed in Table 1, and the comparative results with previous works are displayed in Table 2. In Table 1, three sets of knockout listed are interpreted as the best production of succinate where all the suggested genes were being knocked out. Mutant A shows the removal of *fum* and *zwf* gene resulted in a production of succinate at 6.693 mmol gDW^−1^ h^−1^ and a growth rate of 0.4352 h^−1^. *Fum* represent fumarase, whose function is to reversibly convert fumarate into malate. Removal of *fum* enhances succinate production due to interruption of tricarboxylic acid (TCA) cycle caused by its deletion. As a result, the concentration of fumarate increases, resulting in an increase in succinate production. Furthermore, fumarate is a byproduct of several biosynthetic pathways, and it can only be converted into the desired metabolite succinate [14]. Next, *zwf* gene which converts glucose-6-phosphate (G6P) into ribulose-5-phosphate (Ru5P) and CO2 and generates NADPH that enter the glycolytic pathway for further metabolism. Removal of *zwf* would result in non-operational pentose phosphate pathway and subsequently increase the activity of citric acid cycle [15].

**Table 1:** Knockout lists for succinate in *E. coli*.

Mutant	K/O number	Enzymes	Suggestion	Succinate	Growth
			deletions genes	(mmol/gWD h^−1^)	rate (h^−1^)
A	2	Fumarase (FUM)	*fumA*, *fumB*, *fumC*	6.6930	0.4352
		Glucose-6-phosphate dehydrogenase (G6PDH2r)	*zwf*		
B	3	6-Phosphogluconolactonase (PGL)	*pgl*	6.6930	0.4352
		Phosphotransacetylase (PTAr)	*pta*		
		Succinate dehydrogenase (SUCDi)	*sdh*		
**C**	**3**	**Phosphogluconate dehydrogenase (GND)**	***gnd***	**7.9015**	**0.2761**
		**Pyruvate dehydrogenase (PDH)**	***aceE, aceF, lpdA***		
		**Succinate dehydrogenase (SUCDi)**	***sdh***		

The highlighted and bold part is the best result.

K/O represents knockout.

**Table 2:** Comparative analysis for succinate production.

Method	Enzyme	Succinate (mmol gDW^−1^ h^−1^)
OptKnock [5]	Pyruvate kinase	6.21
	Acetate kinase or phosphotransacetylase	
	Phosphotransferase system	
MOMAKnock [7]	Succinate dehydrogenase	5.02
	6-Phosphogluconolactonase	
	Uricase	
**BATMOMA**	**Phosphogluconate dehydrogenase (GND)**	**7.90**
	**Pyruvate dehydrogenase (PDH)**	
	**Succinate dehydrogenase (SUCDi)**	

Bold row shows the best result.

Mutant B shows the deletion of *pgl* gene, *pta* gene, and *sdh* gene, led to a 6.6930 mmol gDW^−1^ h^−1^ of succinate production and 0.4352 h^−1^ of growth rate. Hager et al. stated that the removal of *pgl* gene that encodes 6-phosphogluconolactonase inhibits hydrolyzation of 6-phosphogluconolactone (6PGL) into 6-phosphogluconate (6PGC) [16]. As a result, 6PGL can be catalysed back into glucose-6-phosphate (G6P), increasing the amount of G6P, a succinate precursor. Furthermore, removing the *pgl* gene prevents the development of the pentose phosphate pathway’s byproducts. Next, knocking out the *pta* gene, which encodes for phosphotransacetylase, decreases acetate and pyruvate accumulation while slightly increasing succinate production [17]. Finally, removing the *sdh* gene, which is active in producing the subunits of succinate dehydrogenase (SUCDi), would prevent succinate from being converted to fumarate in the citric acid cycle [18]. As a result, the amount of succinate produced by the enzyme fumarate reductase encoded by the *frd* gene is significantly increased by catalysing the conversion of fumarate to succinate.

**Figure 4:**
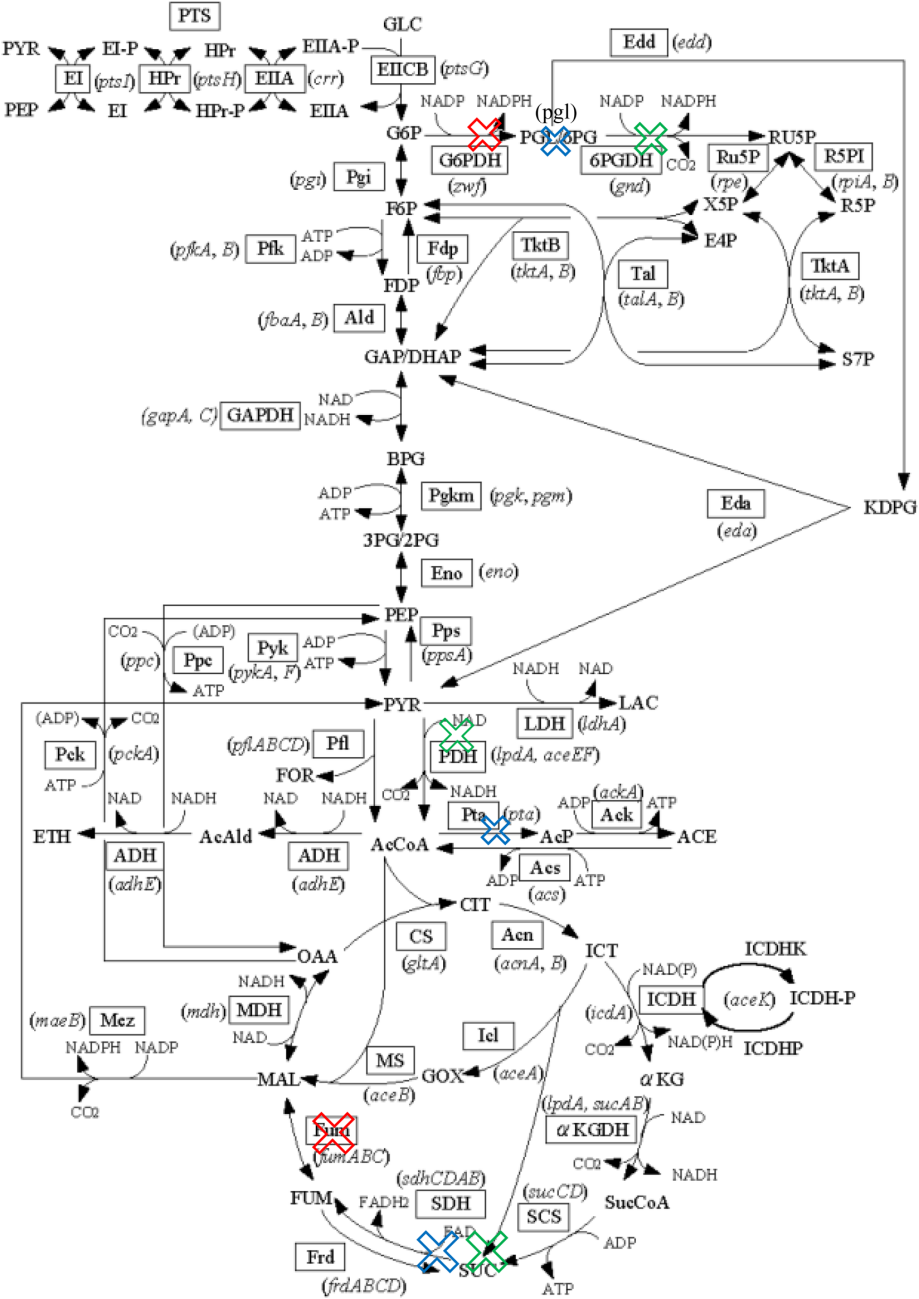
Metabolic pathway of *Escherichia coli* for succinate. The crosses with red, blue, and green color respectively denote mutant A, B, and C, respectively.

Mutant C shows the highest succinate production with 7.9015 mmol gDW^−1^ h^−1^ and 0.2761 h^−1^ of growth rate. Mutant C involved the removal of gnd gene, genes that encode pdh (aceE, aceF, and lpdA), and sdh genes (same as Mutant B). Tang et al. stated that knockout of the *gnd* gene that catalyzes the oxidative decarboxylation of 6-phosphogluconate to ribulose 5-phosphate affects the pentose phosphate cycle [9]. It leads to a large amount of glucose is utilized in the glycolysis and citric cycles to produce more succinate. The removal of *pdh* genes that catalyzes the overall conversion of pyruvate to acetyl-CoA, inhibits pyruvate to be used to produce acetyl-coA. Its removal hence inhibits the formation of competing products such as ethanol and acetate that leads to an increase of succinate production [18]. It acts as the primary link between glycolysis and the tricarboxylic acid (TCA) cycle [19]. By inhibiting the production of competing products, the production of succinate is notably increased. Figure 4 illustrates the succinate pathway of mutant A, B, and C where the genes are being knocked out.

**Figure 5:**
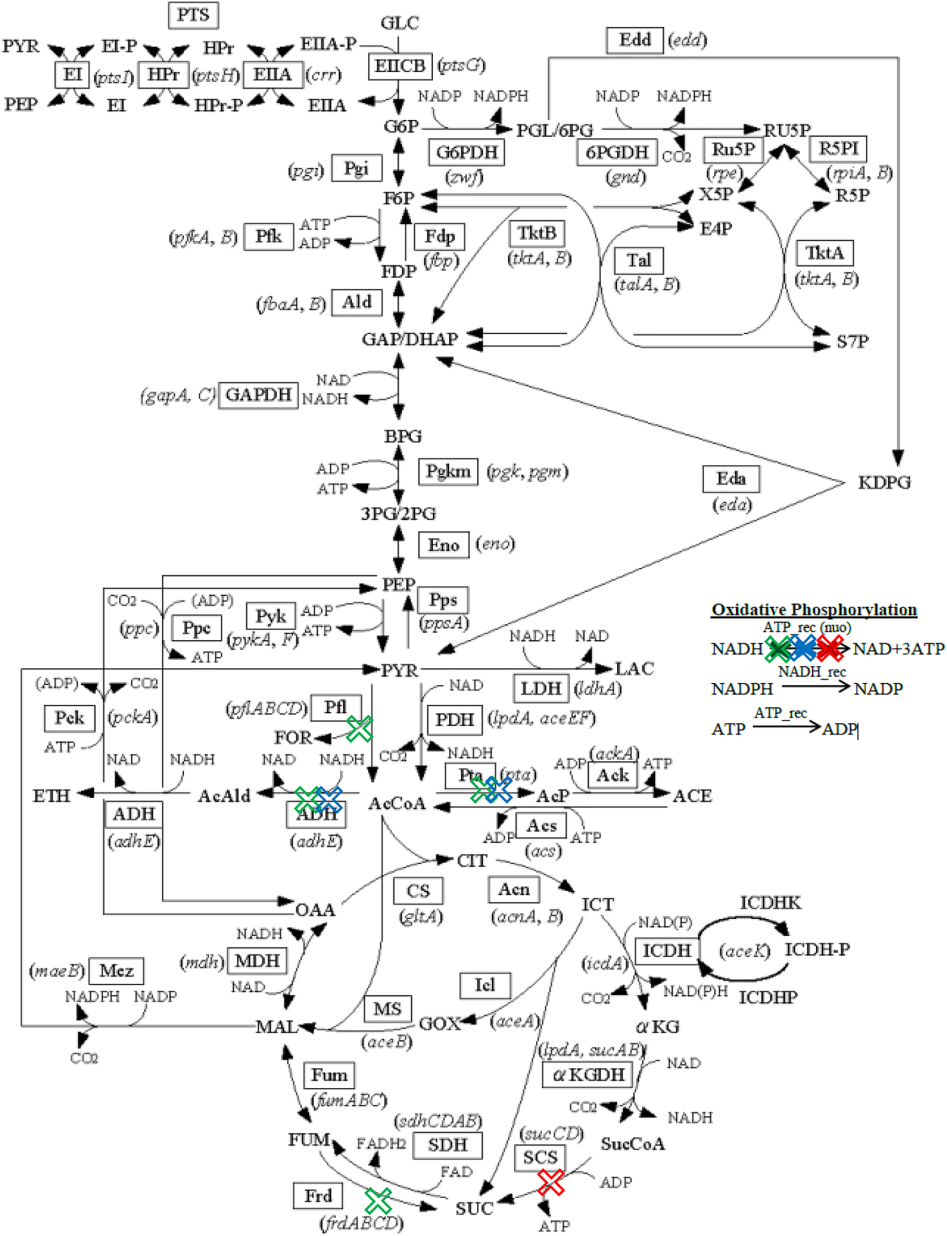
Metabolic pathway of *Escherichia coli* for lactate. The crosses with red, blue, and green color respectively denote the first, second, and third set of gene knockout list respectively.

The proposed algorithm’s production rate is compared to previous works to check if it will generate improved results [5, 6, 9]. A comparison of the findings is seen in Table 2. The removal of pyruvate kinase, acetate kinase or phosphotransacetylase, and the phosphotransferase mechanism, according to OptKnock, results in 6.21 mmol gDW^−1^ h^−1^ succinate production. By knocking out succinate dehydrogenase, 6-phosphogluconolactonase, and uricase, MOMAKnock achieves a production rate of 5.02 mmol gDW^−1^ h^−1^ [5]. Of the three methods, BATMOMA produces the most succinate, with a yield of 7.90 mmol gDW^−1^ h^−1^.

### 3.2 Experimental and comparative results for lactate

After 50 individual runs, Table 3 shows the three knockout lists. *SucCD* and *nuo* genes are knocked out in mutant D, which produce 4.5240 mmol gWD^−1^ h^−1^ and 0.1057 h^−1^, respectively. While the growth rate of *E. coli* is considered high in cases of double gene knockout, the lactate production rate is comparatively poor. Yu et al. explained that sucCD gene products are intermediates in the synthesis of succinate and succinyl-CoA, so they can be eliminated independently [20]. The *nuo* gene then codes for the subunits of NADH dehydrogenase type I (energy-conserving), which is an enzyme that transforms nicotinamide adenine dinucleotide from its reduced to oxidised state. The absence of the gene inhibits aerobic growth in central carbon metabolism, which in turn inhibits citric cycle activity. When the *nuo* gene is mutated, higher yields of d-lactate are produced relative to the wild type strain, according to Yun et al. [21].

**Table 3:** Knockout lists for lactate in *E. coli.*

Mutant	K/O number	Enzymes	Suggestion	Lactate	Growth
			deletions genes	(mmol/gWD h^−1^)	rate (h^−1^)
D	2	SUCOAS	*sucCD*	4.5240	0.1057
		NADH dehydrogenase (NADH16)	*nuo*		
E	3	Acetaldehyde dehydrogenase (ACALD)	*adhE*	11.34	0.1558
		NADH dehydrogenase (NADH16)	*nuo*		
		Phosphotransacetylase (PTAr)	*pta*		
**F**	**5**	**Alcohol dehydrogenase (ACALD)**	***adhE***	**16.1379**	**0.1283**
		**NADH dehydrogenase (NADH16)**	***nuo***		
		**Phosphotransacetylase (PTAr)**	***pta***		
		**Pyruvate dehydrogenase (PFL)**	***pflB***		
		**Fumarate reductase (FRD7)**	***frd***		

The highlighted and bold part is the best result.

K/O represents knockout

The deletion of the *adhE* gene, *nuo* gene, and *pta* gene in Mutant E raises lactate production to 11.34 mmol gWD^−1^ h^−1^ with a growth rate of 0.1558 h^−1^. Since acetyldehyde dehydrogenase is responsible for the inter-conversion of acetyl-coA (ACCOA) and acetaldehyde (ACALD), the precursor of ethanol, the elimination of the *adhE* gene, which encodes acetyldehyde dehydrogenase, reduces ethanol production. Lactate production is enhanced even further by reducing ethanol and acetate production, as all byproducts are eliminated. Since acetyl-coA backflow, phosphoenolpyruvate (*PEP*) and pyruvate (*PYR*) are secreted. *PYR* can also be used to produce lactate by the enzyme lactate dehydrogenase.

The *nuo* gene, as stated in Mutant D, improves lactate production by limiting citric cycle activities by decatalyzing NADH to NAD+. Finally, the *pta* gene, which encodes for the enzyme phosphotransacetylase, was found to be essential in the synthesis of acetate [22]. This gene reduces the ability to produce acetate, which is one of the byproducts. By knocking out these genes, a higher yield of lactate is generated. The *adhE* gene, *nuo* gene, *pta* gene, *pflB* gene, and *frd* gene are all knocked out in Mutant F, and this mutant has the highest production rate and growth rate, respectively, of 16.1379 mmol gWD^−1^ h^−1^ and 0.1283 h^−1^. According to Nikel et al., the *adhE* gene transforms acetyl-CoA to ethanol by three activities: *ADH*, *ACDH*, and *PFL-deactivase*. With the *adhE* gene removed, the production of lactate is greatly increased [23]. Meanwhile, Mutant D and Mutant E demonstrate the implications of deleting the *nuo* and *pta* genes. Next, by inhibiting the *pflB* gene, which catalyses the reversible conversion of pyruvate and coenzyme-A into formate and acetyl-CoA, lactate secretion is increased due to the elimination of competing products [24]. Furthermore, since the *frd* gene’s role is to produce succinate, knocking it out improves lactate production compared to Mutant E. By extracting competing products such as succinate, ethanol, and acetate, Mazumdar et al. claims that lactate production can be increased [25]. As previously mentioned, the *pta* and *adhE* genes inhibit the synthesis of ethanol and acetate, while the frd gene deactivates the synthesis of succinate. The *frd* gene converts fumarate to succinate and reduces dihydrofolic acid to tetrahydrofolic acid. The accumulation of succinate is inhibited when the frd gene is knocked out. By deleting the *adhE* gene, *nuo* gene, *pta* gene, *pflB* gene, and *frd* gene, lactate production is increased while production of competing products such as ethanol, acetate, and succinate is decreased. Figure 5 illustrates the lactate pathway of mutant D, E, and F where the genes are being knocked out.

OptKnock and a hybrid algorithm named Artificial Bee Colony with minimization of metabolic adjustment (ABCMOMA) [9] were used to compare BATMOMA’s lactate production results. By knocking out four genes, phosphofructokinase or fructose-1,6-biphosphate aldolase, acetaldehyde dehydrogenase, phosphotransacetylase, and glucokinase, OptKnock reports a production rate of 10.53 mmol gDW^−1^ h^−1^ of lactate [5]. By knocking out three genes, the *nuo* gene, the *pta* gene, and the *adhE* gene, ABCMOMA produces lactate at a rate of 11.06 mmol gDW^−1^ h^−1^. Since the production of competing products like acetate and ethanol is reduced as a result of this knockout, lactate production increases. The highest lactate production expected by BATMOMA is 16.1379 mmol gDW^−1^ h^−1^, which is the highest among the approaches.

From Tables 2 and 4, BATMOMA shows the highest production rate for both succinate and lactate among the methods. This can be concluded that the hybrid algorithm utilizes the strength of an optimization technique and the advantage of MOMA in identifying a better list of genes to knock out and predicts a better production of succinate and lactate. According to the results, the genes that are successfully identified by BATMOMA is also validated with the biological works.

**Table 4:** Comparative analysis for lactate production.

Method	Enzyme	Lactate (mmol gDW^−1^ h^−1^)
OptKnock [5]	Phosphotransacetylase	10.53
	Phosphofructokinase or fructose-1,6-biphosphate aldolase	
	Acetaldehyde dehydrogenase	
	Glucokinase	
ABCMOMA [9]	NADH dehydrogenase (NADH16)	11.06
	Phosphotransacetylase (PTAr)	
	Alcohol dehydrogenase (ACALD)	
**BATMOMA**	**Alcohol** d**ehydrogenase (ACALD)**	**16.1379**
	**NADH** d**ehydrogenase (NADH16)**	
	**Phosphotransacetylase (PTAr)**	
	**Pyruvate dehydrogenase (PFL)**	
	**Fumarate reductase (FRD7)**	

Bold represents the best result.

### 3.3 Performance evaluation

The mean of BATMOMA’s performance evaluation for succinate production is in the range of 0.41–0.55, with a standard deviation of 0.10–0.16. The mean for lactate production performance is between 0.12 and 0.22, and the standard deviation is between 0.10 and 0.25, which is close to zero. This implies that the lower the dispersion of the growth rate, the closer the value is to zero; it also implies that the data appears to be very close to the mean. Since the variation between the outcomes for each independent run is negligible, these statistical results show that the hybrid algorithm is very stable.

The accuracy of valid solutions is also presented in this paper. It is important to know if a solution is valid as only the valid solution will be taken into account for laboratory experiments. The hybrid algorithm shows if the result is a valid solution or an unsound solution for each run. The result defines a ‘valid’ solution if the value is less than 0.001 whereas the result shows an ‘unsound’ solution if the value is more than 0.001. The accuracy of valid solutions is then calculated by dividing the cumulative counts of ‘valid’ solutions by the total number of runs. After successful evolution to optimum growth rate, the valid solution states that the mutant with the required phenotype (with gene knockouts) will ensure the production of the targeted metabolite, such as succinate and lactate, and would not secrete the other equivalent product. For both succinate and lactate, the accuracy of valid solutions is high as all the values after calculated are less than 0.001 and regarded as valid.

To check if the solution is optimal, a solver status is drawn. Each run has a unique solver status (stat) in a standardised format. Only the solver status of the best population is mentioned in this paper. Gurobi solver version 5.6.2 is used as the solver in this paper. Gurobi solver also supports quadratic programming. As a result, in order to run MOMA in the MATLAB environment, it was decided to include a quadratic programming solver. The accuracy of optimal solution is totally optimal and finest in both succinate and lactate production. Tables 5 and 6 presents that the results of the three performance measurements applied in succinate for each gene knockout after runs for 50 times each.

**Table 5:** Performance measurement of succinate production.

Maximum K/O	Mean	Standard deviation	Accuracy	Accuracy
	(growth rate)	(growth rate)	(valid solution)	(optimal solution)
KO = 1	0.416082	0.158944	$\frac{50}{50}\times 100=100\%$	$\frac{50}{50}\times 100=100\%$
KO = 2	0.466012	0.138097	$\frac{50}{50}\times 100=100\%$	$\frac{50}{50}\times 100=100\%$
KO = 3	0.484110	0.148967	$\frac{50}{50}\times 100=100\%$	$\frac{50}{50}\times 100=100\%$
**KO = 4**	**0.546262**	**0.101380**	$\frac{50}{50}\times 100=100\%$	$\frac{50}{50}\times 100=100\%$
KO = 5	0.529168	0.158389	$\frac{50}{50}\times 100=100\%$	$\frac{50}{50}\times 100=100\%$

The bold represents the best result.

**Table 6:** Performance measurement of lactate production.

Maximum K/O	Mean	Standard deviation	Accuracy	Accuracy
	(growth rate)	(growth rate)	(valid solution)	(optimal solution)
KO = 1	0.162610	0.139981	$\frac{50}{50}\times 100=100\%$	$\frac{50}{50}\times 100=100\%$
**KO = 2**	**0.133434**	**0.102494**	$\frac{50}{50}\times 100=100\%$	$\frac{50}{50}\times 100=100\%$
KO = 3	0.129124	0.102539	$\frac{50}{50}\times 100=100\%$	$\frac{50}{50}\times 100=100\%$
KO = 4	0.154272	0.168284	$\frac{50}{50}\times 100=100\%$	$\frac{50}{50}\times 100=100\%$
KO = 5	0.218196	0.259452	$\frac{50}{50}\times 100=100\%$	$\frac{50}{50}\times 100=100\%$

The bold represents the best result.

## 4 Conclusion and future work

In short, this paper presents a new hybrid algorithm named BATMOMA to identify the list of genes to delete in order to enhance the production of the target chemical. The results show that BATMOMA performs better among the methods in predicting succinate and lactate production by identifying a more suitable set of genes to be knocked out. BATMOMA has the advantage of performing both global and local searches simultaneously and multivariable function optimization. The Bat algorithm is an accurate and very efficient algorithm to solve complex problems. The nature of automatic zooming, effective parameter control, frequency turning, and echolocation are great things to solve a wide range of problems with quick time in promising optimal solutions. MOMA is a promising method that gives a better understanding of flux states through distance minimization in flux space and particularly computing the fitness of a specific gene knockout.

However, the disadvantage of Bat algorithm is, it converges very quickly at an early stage, and also convergence rate will slow down. In large scale applications, the accuracy is limited, and no defined mathematical analysis links the parameters with convergence rate. Hence, Bat algorithm can be further improved to solve these two problems. BATMOMA can be used to test on different datasets such as *Clostridium acetobutylicum*, *Chlamydomonas reinhardtii*, and *Saccharomyces cerevisiae* that are recently used in biofuels production [26–28]. Besides, BATMOMA can also be improved in the future by doing multi-objectives such as maximizing the production and growth rate and running the whole genome model with minimum computational time.
